# Differences of patients’ characteristics in acute type A aortic dissection – surgical data from Belgian and Japanese centers-

**DOI:** 10.1186/s13019-018-0782-x

**Published:** 2018-09-04

**Authors:** Motohiko Goda, Tomoyuki Minami, Kiyotaka Imoto, Keiji Uchida, Munetaka Masuda, Bart Meuris

**Affiliations:** 1 Department of Cardiac Surgery, University Hospitals Leuven, Leuven, Belgium; 2 Cardiovascular Center, Yokohama City University Medical Center, Yokohama, Japan; 3 Department of Cardiovascular Surgery, Yokohama City University Hospital, Yokohama, Japan

**Keywords:** Acute aortic dissection, Epidemiology, Gender differences

## Abstract

**Background:**

It is well known that there are major differences between the Japanese and Western population regarding the incidence of ischemic heart disease and stroke. The purpose of this study was to evaluate differences of patients’ characteristics between Belgian and Japanese cohort with acute type A aortic dissection.

**Methods:**

In 487 patients (297 male patients, mean age 61.9 ± 12.2 yrs) who underwent surgery for acute type A aortic dissection, baseline preoperative and intraoperative data were collected. Belgian patients (*n* = 237) were compared to Japanese patients (*n* = 250). Clinical data included patient demographics, history, status at presentation, imaging study results and intraoperative findings.

**Results:**

The Japanese cohort had significantly more women (48.8% vs. 28.7%, *p* < 0.0001), lower BMI (24.2 vs. 26.4, *p* < 0.0001) and lower prevalence of hypertension (49.2% vs. 65.8%, *p* = 0.0002). More DeBakey type I dissections and less type III dissections with retrograde extension were reported in Belgium than in Japan (77.2% vs. 48.4%, *p* < 0.0001, 3.4% vs. 38.7%, *p* < 0.0001, respectively). More entries were found in the ascending aorta (78.5% vs. 58.5%, *p* < 0.0001) and aortic arch (24.9% vs. 13.7%, *p* = 0.0018) in Belgian patients than in Japanese patients, who had more entries in the descending aorta or undetected entries.

**Conclusions:**

In acute type A aortic dissection, Belgian patients reveal striking differences from Japanese patients regarding gender distribution, entry tear location and type of dissection. Japanese women are more likely to develop acute type A aortic dissection than Belgian women. (234 words).

## Background

It is well known that the prevalence of ischemic heart disease and its mortality are significantly lower in Japan compared to the United States or to other Western populations [1–4]. On the contrary, Japanese people suffer significantly more from stroke, mainly the hemorrhagic type of stroke [1–4]. Within these two major cardiovascular disease types, there is a clear East/West divide [1]. Next to obvious and important differences in dietary habits (lower saturated fat and higher salt intake for the Japanese with subsequently much lower serum cholesterol levels), there might be genetic differences between Japanese and Western people that underlie the observed differences in cardiovascular diseases [1–4]. But the actual causes for these differences in cardiovascular risk profile remain unknown and any explanation remains speculative. There is a higher prevalence of arterial hypertension within the general Japanese population, but this is insufficient to explain all phenomena. And although the Japanese dietary pattern has westernized these days, the trends in cardiovascular risk still last [4].

Acute type A aortic dissection (TA-AAD) is a life-threatening cardiovascular catastrophe which often requires emergency surgery to prevent death [5]. Within the International Registry of Acute Aortic Dissection (IRAD), geographic differences in clinical presentation, treatment and outcome in type A dissection have been studied, comparing European patients to North Americans. This study was able to reveal small differences in presentation and methods used for diagnosis, but overall these two Western patient cohorts were quite comparable [6]. Despite the known and described differences in ischemic heart disease and stroke between Asian and Western people, a comparison within patients with type A dissection has not been performed yet. The purpose of the present study is to evaluate differences in baseline patient characteristics and TA-AAD profile between Belgian and Japanese patient cohort.

## Methods

### Study population

We collected data from 487 patients (297 male; mean age 61.9 ± 12.2 y; range 14–88 y) with TA-AAD who underwent emergency surgery at a Belgian center (University Hospital Leuven, Leuven, Belgium) or at a Japanese center (Yokohama City University Medical Center, Yokohama, Japan). Surgical interventions were performed in a time frame between January 1986 to September 2011 in the Belgian center and from January 2003 to December 2010 in the Japanese center. Diagnosis was made by imaging studies including computed tomography, magnetic resonance imaging and/or echocardiography. TA-AAD was defined as any dissection that involved the ascending aorta with presentation within 14 days of its onset. Approval by the local ethics committee was granted to collect and analyze patient data.

### Data collection

Baseline clinical data on enrolled patients, including patient demographics, medical history, clinical findings at presentation, imaging study results, preoperative complications and intraoperative findings were collected and stored by retrospective chart review. Body mass index (BMI) was calculated (weight(kg)/height(m)^2^).

### Literature and cardiac surgical society data

In order to cross-check the baseline characteristics from our study cohort with current literature, a Pubmed search was performed for recent reports on TA-AAD within either Western centers (Europe, USA) or Eastern centers (Japan, Korea). Also, recent data on TA-AAD incidence from both the Belgian [6] and Japanese [7] cardiac surgical societies were used to estimate nationwide absolute incidence values.

### Statistical analysis

All statistical analyses were performed using StatView J-5.0 (SAS Institute Inc., Cary, NC). Continuous variables were expressed as mean ± standard deviation and were compared using the student’s *t* test. Categorical variables are expressed as absolute numbers or percentages and were compared using chi-square testing. The level of statistical significance was set at *p* < 0.05.

## Results

### Demographics and patient history

Of the 487 patients enrolled in this study, 237 were from Belgium, and 250 were from Japan. All studied variables are listed in Tables 1 and 2. The proportion of women was significantly higher in Japan than in Belgium (48.8% vs. 28.7%, *p* < 0.0001). Belgian women were younger than Japanese women at the time of operation (61.2 vs. 67.6y, *p* = 0.0006). Belgian patients had a higher BMI (26.4 vs. 24.2, *p* < 0.0001), they were taller (men; 1.76 cm vs. 1.68 cm, *p* < 0.0001, women; 1.64 cm vs. 1.54 cm, *p* < 0.0001), and heavier (men; 82.5 kg vs. 72.2 kg, *p* < 0.0001, women; 70.2 kg vs. 54.5 kg, *p* < 0.0001) than Japanese patients.

**Table 1:** Demographics and patient history for Belgian and Japanese patients

Variables	Belgium (*n* = 237)	Japan (*n* = 250)	*p* Value
Gender (Men/Women)	169/68	128/122	< 0.0001
Age (yrs)	59.3 ± 12.7	63.3 ± 11.3	< 0.0001
Age < 40 yrs	13 (5.5%)	5 (2%)	0.0416
Age ≥ 80 yrs	10 (4.2%)	19 (7.6%)	0.1151
Body Mass Index	26.4 ± 4.1	24.2 ± 3.7	< 0.0001
Hypertension	156 (65.8%)	123 (49.2%)	0.0002
Known aortic aneurysm (≥ 4 cm)	51 (21.5%)	149 (59.6%)	< 0.0001
Bicuspid aortic valve	5 (2.1%)	1 (0.4%)	0.1008
Marfan syndrome	7 (3%)	7 (2.8%)	0.9192
Other connective tissue disease	2 (0.8%)	0 (0%)	0.1455
Pregnant	2 (0.8%)	0 (0%)	0.1455
Drug	2 (0.8%)	0 (0%)	0.1455
Trauma	1 (0.4%)	0 (0%)	0.3039
Iatrogenic	4 (1.7%)	0 (0%)	0.0391
Previous cardiac operation	15 (6.3%)	4 (1.6%)	0.0071
Post cardiac operation within hospital stay	3 (1.3%)	0 (0%)	0.0744
Post cardiac operation within 2 years	13 (5.5%)	1 (0.4%)	0.0008

**Table 2:** Demographics by gender

Variables	Belgium	Japan	*p* Value
Men	*n* = 169	*n* = 128	
Age (yrs)	58.6 ± 11.8	61.1 ± 11.1	0.0607
Age < 40 yrs	8 (4.7%)	3 (2.3%)	0.2801
Age ≥ 80 yrs	6 (3.6%)	5 (3.9%)	0.8722
Length (m)	1.75 ± 0.07	1.68 ± 0.06	< 0.0001
Weight (kg)	82.5 ± 14.1	72.2 ± 11	< 0.0001
Body Mass Index	26.5 ± 3.9	25.5 ± 3.4	0.0166
Hypertension	107 (63.3%)	62 (48.4%)	0.0104
Women	*n* = 68	*n* = 122	
Age (yrs)	61.2 ± 14.5	67.6 ± 10.5	0.0006
Age < 40 yrs	5 (7.4%)	2 (1.6%)	0.0045
Age ≥ 80 yrs	4 (5.9%)	14 (11.5%)	0.2059
Length (m)	1.64 ± 0.08	1.54 ± 0.07	< 0.0001
Weight (kg)	70.2 ± 13.6	54.5 ± 10.5	< 0.0001
Body Mass Index	26.5 ± 3.9	25.5 ± 3.4	< 0.0001
Hypertension	49 (72.1%)	61 (50%)	0.0082

Belgian patients had more preoperatively documented arterial hypertension (65.8% vs. 49.2%, *p* = 0.0002), while in Japanese previously documented aortic aneurysms were more prevalent (59.6% vs. 21.5%). Belgian patients had more previous cardiac operations (6.3% vs. 1.6%, 0.0071) than Japanese patients and there were more iatrogenic dissections (1.7% vs. 0%, *p* = 0.0391). There were no significant differences with regard to history of bicuspid aortic valve, Marfan syndrome, pregnancy, drug abuse or trauma.

### Type of aortic dissection and preoperative complications

Table 3 shows that DeBakey type I dissections were more frequent in Belgium (77.2% vs. 48.4%, *p* < 0.0001) while Type III dissections with retrograde extension were less frequent in Belgium than in Japan (3.4% vs. 38.7%, *p* < 0.0001). More entries were found in the ascending aorta (78.5% vs. 58.5%, *p* < 0.0001) and aortic arch (24.9% vs. 13.7%, *p* = 0.0018) in Belgian patients than in Japanese patients.

**Table 3:** Type of aortic dissection and complications for Belgium and Japanese patients

Variables	Belgium (*n* = 237)	Japan (*n* = 250)	*p* Value
DeBakey I	183 (77.2%)	121 (48.4%)	< 0.0001
DeBakey II	43 (18.1%)	31 (12.4%)	0.084
Stanford type B with retrograde extension	8 (3.4%)	96 (38.4%)	< 0.0001
Entry			
Ascending aorta	186 (78.5%)	145 (58%)	< 0.0001
Aortic arch	59 (24.9%)	34 (13.6%)	0.0018
Descending aorta	8 (3.4%)	25 (10%)	0.0084
Not found	0 (0%)	44 (17.6%)	< 0.0001
Shock	67 (28.3%)	47 (18.8%)	0.0136
Cardiac tamponade	41 (17.3%)	74 (29.6%)	0.0014
Cardiopulmonary resuscitation	6 (2.5%)	14 (5.6%)	0.0881
Stroke	16 (6.8%)	16 (6.4%)	0.8758
Paraparesis/paraplegia	9 (3.8%)	2 (0.8%)	0.0261
Lower limb ischemia	27 (11.4%)	30 (12%)	0.8349
Renal ischemia	13 (5.5%)	3 (1.2%)	0.008
Mesenteric ischemia	4 (1.7%)	9 (3.6%)	0.1907
Myocardial ischemia	15 (6.3%)	13 (5.2%)	0.5926

Shock was more frequent in Belgian patients than in Japanese patients (28.3% vs. 18.8%, *p* = 0.0136), however, cardiac tamponade was more frequent in Japan than in Belgium (29.6% vs. 17.3%, *p* = 0.0014). As to complications associated with impaired arterial blood flow, the prevalence of renal ischemia (5.5% vs. 1.2%. *p* = 0.008) and paraparesis/paraplegia (3.8% vs. 0.8%, *p* = 0.0261) were more frequent in Belgium than in Japan. There were no significant differences with regard to development of stroke, myocardial ischemia, mesenteric ischemia, and limb ischemia.

## Discussion

The occurrence of cardiovascular diseases is certainly associated with genetic factors, life style, dietary factors, environmental conditions and coexisting diseases. Therefore, it is reasonable that there are regional differences in incidence and mortality of different cardiovascular pathologies. The fact that Japan has less ischemic heart disease and more hemorrhagic stroke has been epidemiologically studied and is well recognized these days [1–4, 8, 9]. In the present study we highlight that also acute type A dissection is a disease with regional differences in patient characteristics when Belgian and Japanese patient cohort are compared.

The larger proportion of women in Japanese TA-AAD patients is a remarkable demographic finding in this study. Based on reports from predominantly Western centers, the gender distribution in TA-AAD has always been described as a 2:1 male:female ratio [10–12]. This 2:1 male/female ratio is frequently reported by IRAD and can be found in many textbooks of cardiac surgery [13, 14]. But does this ratio hold true for the Eastern population? The Japanese series in this study showed 48.8% female patients (who were also significantly older). Other reports from Japanese centers (with at least 80 surgical TA-AAD cases) also reported as many as 39.1 to 58.8% of female patients [15–20]. Similarly, recent reports from Korea [21–24] described proportions of female patients up to 42.4 to 52.7%, very similar to the reports from Japan. Therefore, it seems that within the East-Asian population, the gender distribution male:female in TA-AAD patients is more a 1:1 ratio, or Asian women are more likely to develop TA-AAD compared to Western women. According to the organization for economic co-operation and development (OECD) [25], the number of annual deaths from circulatory system diseases in Japanese women was 380 per 1,000,000 persons per year, which is much less than the 940 deaths per million per year in Belgian women. With a significantly lower overall death from cardiovascular diseases, it is paradoxical that Japanese women are more likely to develop TA-AAD, but the etiology behind it might be similar to that causing the higher numbers of hemorrhagic stroke in Japanese women [4]. One can hypothesize that Japanese woman have a more pronounced vessel wall fragility.

The detected differences in the incidence of arterial hypertension are paradoxical, because Japanese have a higher nationwide prevalence of hypertension [25]. Also the fact that more Japanese had previously documented aneurysms is enigmatic. The remaining observed differences in number of redo cases, iatrogenic cases, occurrence of malperfusion and tamponade should be interpreted with caution, given the relatively low number of observations.

It is impossible to investigate the absolute incidence or prevalence of acute aortic dissection. Reported incidences may underestimate the ‘real’ population value, because TA-AAD also causes sudden death before any diagnostic investigation has been performed. Textbooks teach us an ‘estimated worldwide incidence’ of 5 to 29.5 per 1,000,000 per year [26]. But also figures between 29 to 52 per 1,000,000 per year have been reported [27–30]. It is important to know that some of these values originate from observations of relatively small patient cohorts and are then extrapolated to the general population of a country. Current data from two Western cardiac surgical societies and registries teach us the following incidences per 1,000,000 persons per year: Belgium 8.1 [7]. The IRAD registry reports an annual incidence of 4.1 per million and data from the USA show an estimated incidence of 2 to 8 per million per year [26]. Surprisingly, Japan reports 27.2 operated TA-AAD cases per million persons in 1 year [31], which is 4 to 7 times higher compared to the current Western data. It seems that the worldwide incidence of acute aortic dissections is indeed - like the textbooks show - within a range 5 to 30 per million per year, but that the incidence in Western people is more on the lower side of this spectrum while Japanese people are on the higher side.

Within TA-AAD patients, type I dissections are the predominant form (in Western series), and in up to 60–68% of cases, the location of the entry tear is within the ascending aorta [13, 14, 26]. Our observation confirmed this pattern and the main entry tear location. But the Japanese data show a different pattern: fewer entries were found in the ascending aorta and more retrograde dissections extending from the descending aorta were observed. Also in a high proportion of patients, no entry tear was found at all, suggesting a tear in the lower descending aorta. The retrograde extension of aortic dissection leads patients with type B dissection to type A. This may partly explain the higher number of TA-AAD cases in Japan compared to other western countries.

Both more prevalent of documented aortic aneurysm and the retrograde extension of aortic dissection are possibly attributed to a vessel fragility of Japanese people. The reason why there would be higher vessel fragility in Japanese remains unknown. Hypotheses have been provided linking low serum cholesterol levels to altered mechanical properties of endothelium and vessel wall quality. Further investigation with comparison of biomechanical characteristics of tissue within both populations is needed to elaborate our findings further.

## Conclusion

Belgian patients with TA-AAD differ from Japanese patients with respect to gender, entry tear location and type of dissection. The absolute incidence of TA-AAD in Japan is higher. Especially Japanese women are more likely to develop TA-AAD than Belgian women.

### Study limitations

This study included single center data from each country and the numbers of the subjects are relatively small. Only patient data with TA-AAD were evaluated in this study, medically treated cases, chronic dissections or type B dissection data was not included. Pre- and intraoperative factors alone were gathered and studied, without outcome data. The study periods were different between the two centers. No pathological or biomechanical investigations were performed.

## Abbreviations

BMI: Body mass index
IRAD: The International Registry of Acute Aortic Dissection
OECD: the organization for economic co-operation and development
TA-AAD: Acute type A aortic dissection
